# Phase II investigation of a PARP inhibitor (olaparib) in castration resistant prostate cancer (CRPC) which incorporates the possibility that treatment effect may be restricted to biomarker defined subgroups

**DOI:** 10.1186/1745-6215-12-S1-A88

**Published:** 2011-12-13

**Authors:** Roger A’Hern, Johann DeBono, Shahneen Sandhu, Eletheria Kalaitzaki, Martine Usdin, Emma E Hall

**Affiliations:** 1 Clinical Trials and Statistics Unit, Institute of Cancer Research, Sutton, Surrey, SM2 5NG, UK; 2 Department of Experimental Cancer Medicine, Institute of Cancer Research, Sutton, Surrey, SM2 5NG, UK

## Objective

To undertake a phase II trial to identify the best CRPC patient group(s) to be studied for sensitivity to olaparib in a randomised, controlled phase III trial.

## Methods

The TO-PARP trial has a multistage phase II design consisting of a non-randomised component with response as the primary endpoint followed by a randomised component with overall survival as the primary endpoint.

Non-randomised component : This part of the trial design allows rapid progression to a randomised comparison if there is evidence of a high response rate (50% or more) in unselected patients. If the evidence for a high response rate in unselected patients is weak, biomarker defined groups will be investigated for their sensitivity to olaparib.

The first stage will include 30 CRPC patients. If 15 (50%) or more respond then no more patients will be entered and the randomised component will be undertaken in unselected patients. Should 2 (7%) or fewer respond then olaparib will be rejected. If between 3-14 (10%-47%) patients respond then a further 15 patients will be entered. Should 23 (51%) or more respond overall then the randomised component will be undertaken in unselected patients and if 5 (11%) or fewer respond then olaparib will be rejected. Otherwise with 6-22 responders (13%-49%), biomarker analysis of tissue collected from all 45 patients will be undertaken. This will aim to identify a sensitive subgroup, with a response rate which is compatible with a 50% response rate, warranting further assessment in the randomised component. If such a subgroup is found, a confirmatory single stage 44 patient trial will be undertaken in this subgroup; this will also serve to pilot prospective biomarker testing in a multi-centre clinical trial setting.

Randomised component : This is a phase II controlled, definitive endpoint assessment of the results generated in the non-randomised part, offering a more secure foundation for efficacy before proceeding to phase III. 180 patients will be randomised 2:1 to olaparib or an appropriate standard of care (α 1-sided 10%, power 80%).

## Conclusions

Randomised phase II trials are the gold standard to establish a basis for treatment efficacy but only a subset of patients may be sensitive to a new therapy. This possibility was incorporated into the design of the TO-PARP trial by introducing a non-randomised initial component with the potential to identify a sensitive subgroup.

